# General Pathology

**Published:** 1843-10

**Authors:** 


### ART. XV.

1. *Allgemeine Krankheitslehre.* Von Dr. K. F. H. MARX, ordentlichem Professor der Medicin in Göttingen, &c.—*Göttingen*, 1833. 8vo, pp. 273.

*General Pathology.* By Dr. K. F. H. MARX, Ordinary Professor of Medicine at Göttingen.—*Göttingen*, 1833.

2. *Grundzüge zur Lehre von der Krankheit und Heilung.* Von Dr. K. F. H. MARX, &c.—*Carlsruhe und Baden*, 1838. 8vo, pp. 447.

*Elements of Pathology and Therapeutics.* By Dr. K. F. H. MARX, &c.—*Carlsruhe and Baden*, 1838.

THE medical sects of Germany may be arranged in four or five principal divisions: First, there is the metaphysical, fanciful, and purely theoretical school, peculiar almost to Germany, and giving its hue to all the rest. Second, the physiological or biological. Third, the bio-chemical or material. Fourth, the Hippocratic, empirical, or expectant. To the first division belong the Kantian physicians, the homœopathists, hydriatics, Brownists, and all who deduce details from some general principle or principles, including a host of philosophers whose principles are dreams and visions. Those in the second division, without neglecting chemistry and natural physiology, seek in philosophical anatomy and the laws of transcendental physiology for the true basis of pathology. Schultz, whose work is reviewed in our last Number, is an example of this school. The third is nearly allied to the preceding, but differs in making physiology subservient to organic chemistry and natural physiology. Liebig is the type of this class. The fourth is rather learned than scientific, and is professedly eclectic. The results of the science and observation of past times are not neglected although old, nor are the researches of the moderns despised because they are new. The physicians of this class are, however, rather obstructive than progressive, rather readers than doers; and more inclined to analytical criticism than to experiment. Göttingen may be considered the head-quarters of the fourth class, central Germany of the third, Berlin of the second, and southern Germany, especially the secondary schools, of the first. The Göttingen school approaches the nearest of any to the English, being less outrageously speculative, and more inclined to matter of fact than the others. We speak, of course, generally, for we are quite aware that there are many zealous cultivators of medical science in every part of Germany, who are as practical and as averse to mysticism as ourselves.

The perusal of the works of Professor Marx, and our own knowledge of his intellectual character, would induce us to place him in the fourth of the divisions we have just mentioned. His *Origines Contagii*, and his critical analyses of the doctrines and writings of Herophilus and Paracelsus show very great literary attainments, and an astonishing extent of medical reading; while the works before us exhibit the results of the peculiar line of research into which the intellectual tastes and habits of the author have led him.

The work first mentioned contains a concise and systematic view of the various theories of medicine extant, and comprehensive definitions of the technical terms to which those theories have given origin. It is professedly elementary, being intended for the use of his own class, and as an introduction to the study of medicine. It contains much information in little compass, and is carefully and accurately written. We would strongly recommend its perusal to the more advanced English student who wishes to improve himself in the German language, and at the same time acquire, at little cost of time or money, a clear and exact knowledge of medical theories unencumbered by digressive details,

The Principles of Pathology and Therapeutics is a work of higher pretensions than the preceding, being written for the accomplished physician, and professing to bring the fundamental doctrines of medicine into connexion with the most recent anatomical, physiological, and pathological researches, and with the results of the allied sciences. In doing this, our author professes to avoid all merely controversial topics and to exhibit the art of healing to laymen such as it really is, a thing, not of science and experience merely, but of conscience and earnest conviction. The following paragraph from the introduction exhibits the spirit of eclecticism with which our author is imbued. It will be observed that he professes to steer between the doctrines of the first and third school we have already defined.

"Those who look upon the human body as a mere machine kept in motion by chemical affinities and physical forces, feel themselves compelled to seek for the cause of deranged function and death in disordered affinities, or interrupted processes. On the other hand, they who contemplate the body as being only the outward and visible part of the soul,—as the symbol of spiritual force,—ascribe interrupted function to disorder of the inner life;—to a continuous alternation of relations between the inner exciting force and its external results;—to an impaired action of the soul on the body;—to a yielding of the will, the feelings, and the thoughts to matter; or to a perverted, or a too strong or too weak condition of the nervous system, the medium of all action. Each party seeks to explain the phenomena of disease according to its own favorite views, and feels obliged to develop the guiding ideas in logical coherence with the facts to be explained. This strife is equally natural and praiseworthy. But the fancies of individuals cannot be considered as genuine science, for genuine science is simply the result of ideas clearly demonstrated and communicated. Notions assumed without proof peril its very existence, or at least, subjective opinions must be considered as far inferior in value to objective certainty, so that science necessarily refuses to receive mere probabilities or beliefs within its pale, and justly accepts inductions from facts only." (p. 9.)

In looking over the early pages of our author's volume, we remark less of that severe induction and adherence to facts than might have been expected. Real facts, and especially general facts, are yet *desiderata* in medicine. The plan and scope of his work being an exposition of principles, may, perhaps, in some degree account for this, but we think it will not excuse the grave exposition of acknowledged axioms. We know well that the intellectual genius of his nation, (and of this, the grammatical construction of its language is a proof,) is diametrically contrary to our own, and that an exposition of first principles, and not an exhibition of particular facts, is the usual commencement of a German treatise. It is not long since we took up a German writer on the Races of Mankind, and found that he considered it necessary to commence his essay with a definition and analysis of the Trinity. Nothing so absurd as this is to be found in Professor Marx's work; but he gravely states principles which would have been considered mere truisms by an English writer, addressing himself to the educated practitioner. In considering prophylaxis, for example, he would not have stated in so many words, that a knowledge of the first beginnings and primary links in the chain of morbid causation is requisite to prevention. And in considering hurtful influences and their removal, it is a matter of course that things may be prejudicial to the close student, which are useful, or at least not injurious to the hard-working, unthinking, unanxious rustic. We do not mean to say that every practitioner bears this difference in mind, for some would deplete the sensitive and highly organized man of genius as freely as the clown; but certainly every judicious practitioner remembers it, and treatment to the contrary is rather the result of indolent routinism, than want of proper knowledge. It is true some English writers have found a mare's nest, and published diatribes against injudicious depletion, on the supposition that all the world had been as tardy as themselves in discovering that routinism was not skill. This circumstance can only be said to prove the ignorance of the authors, and not of the educated part of the profession.

Professor Marx's history of the evolution of disease exhibits much thought; and, though the views are not exactly novel, are worthy the prominence given to them, as they are too little considered by the generality. A disease may really commence with conception, but remain dormant until, in the progressive evolution of the body to perfection, or in its gradual decline, the circumstances occur favorable to the manifestation of the inherent morbid action. This inherent predisposition to disease and its results may be seen in a phthisical family, the members of which are cut off in regular succession as they attain a certain age. Paralysis, insanity, and other diseases may be observed to follow the same law of development in families. The following is a specimen of our author's style and matter:

"The hereditary morbid predisposition is apparent at an early age to the scientific observer; to the ignorant it appears only when the time of its outbreak has come, that is to say, when awakened into activity by injurious agencies, or when the organ in which it had laid dormant comes into full action. Hence the inveteracy and incurable nature of the diseases arising from these causes. A disease of this kind is not so simple as it appears. It is as old as the individual, and sympathises with him in all that he passes through. It is not merely as if a seed lay dormant in an organ, the husk of which need only be burst open to allow it to germinate; the whole organism feels its presence, and is too often in danger, not to be aware that it incloses an unfriendly influence. And this unfriendly influence operates more actively than is usually supposed. The development of the body, nay, even the mental organization, is often determined by it." (p. 30.)

In treating of the susceptibility to disease, it is remarked that there is in this respect, as in others, a sympathy and an antipathy, apparently regulated by certain obscure laws of affinity, intermediate in character between the psychical and the physical. Just as a certain degree of temperature determines whether bodies shall combine mechanically or chemically, so in physiology and pathology there must be similarity and dissimilarity, or more properly a sort of polarization. A person will die of smallpox at an advanced age, in whom all attempts to excite the disease in youth had failed. A woman may be many years married and yet childless, but becoming a widow and remarrying to an individual, very similar to or dissimilar from herself, she speedily becomes pregnant. There are other interesting observations under this head we would gladly have cited if our space would have allowed.

The causes of diseases, and their usual divisions into remote and proximate, predisposing and exciting, external and internal, are noticed; as also the influence of the different periods of life and their physiological condition. The fundamental requisites for the cure of disease, the nature and development of the latter, and the general curative indications, are commented on in succession. A considerable space is occupied with an interesting and detailed notice of the predisposing causes of disease, as they are psychical, structural, or physical. The pathology of the passions, temperaments, and idiosyncrasies; of the solids, of the blood, and the secretion, and of the functions of organs; the pathological relations of kosmic and telluric influences, and of air, light, sleep, food, clothing, baths, poisons, contagions, and infectious matters, are all successively reviewed. Under the head of "Time and space in relation to disease," the effects of climate and soil, and the pathology of endemics, the influences of the diurnal periods and of the seasons, and the pathology of epidemics, the stages and periods of diseases, and the doctrine of critical days—are all noticed.

Professor Marx arranges his therapeutical agents in three principal divisions, as they are strengthening, debilitating, or alterative. In the first division the excitant, irritant, tonic, and astringent classes of remedies are considered. In the second we find anodynes and antispasmodics, and the dietetic or moderately antiphlogistic, and the actively antiphlogistic methods of treatment. The third comprises the evacuants, namely, emetics, purgatives, diuretics, diaphoretics, and expectorants, the derivatives, and a class of remedies which may be termed alterative tonics, comprising metallic tonics and alteratives, the hunger-cure, travelling, and spa-ing.

As all this matter is condensed in fewer than 450 pages, any further condensation is impossible; and as so much of it is valuable, we feel an *embarras des richesse* in attempting a selection. The following exposition of the much misunderstood hematophobia of our German brethren will remove some prejudices:

"Blood is drawn in full stream from a vein, or by leeches or cupping-glass from the skin, according to the nature, extent, and intensity of the disease, and the constitution of the patient. The one is termed general, the other local, bloodletting. General bloodletting is to be practised without reference to the age of the patient, when there is inflammation of any important tissue, and a full and hard pulse; or even when the pulse is compressed, provided its smallness is unconnected with any cardiac disease, and leaves no doubt that the central organs of the circulation are overflowing with blood. Local bloodletting is advisable when the seat of the disease is easily accessible; when the inflammatory action is limited; when general bleeding has been already practised; when there is a want of power in the system; or when a cachectic state of the system renders general bleeding dangerous; and when local depletion is alone necessary, as in suppressed constitutional hemorrhages. Cupping affords the same immediate relief in external inflammation, as general bleeding affords in internal, or in visceral congestion. The pressure of the cupping-glass has an influence on the capillary circulation, as has also the bleeding after the removal of leeches. The nearer to a suffering organ blood can be drawn, the better. The so-termed derivative bleedings are more effectual than the revulsive. The quantity of blood proper to be drawn in each case cannot be estimated by the eye or a measure. It must be determined by the symptoms, constitution, and age of the patient, and the nature of the disease. In general, from six to eight ounces is a moderate bleeding for an adult; from twelve to sixteen ounces may be considered a copious abstraction of blood. The loss from a leech-bite may be estimated at from three to four drachms for every half-hour the bleeding continues. The loss by cupping depends on the repetition of the scarification, and the size of the glasses. The bad consequences of a copious loss of blood appear either immediately or in a short time. They are fainting, noises in the ears, dimness of sight and even blindness, sudden startings from sleep, delirium, gasping for air, prostration of strength, convulsions, stupor." (p. 364.)

The general rules for determining the quantity of blood to be drawn are thus laid down:

"The extent to which bloodletting should be practised is determined by the urgency of the symptoms, by the importance of the structure affected, by the number of blood-vessels in the inflamed viscus, by the habits of the patient with regard to bleeding, and by the nature of the disease. The greater the inflammation, the more freely must the blood be taken, allowing it to flow until the hardness and fullness of the pulse be abated and the pain relieved.........If other circumstances indicate bloodletting, the presence of the menses, lochia, or hemorrhoidal flux do not contra-indicate the operation. It is of more importance to consider whether the tissue affected be not intolerant of any considerable loss of blood, as for example, the mucous tissue; or whether the heart itself be not affected, and the indications for bleeding consequently appear more urgent than they really are; or whether the blood itself be not in a state of irritation and expansion, as in puerperal or the bilious fever." (p. 366.)

Our author very correctly observes that nothing can be learnt from the buffy coat as to the necessity for further bleeding. He observes that it is altogether unconnected with the nature of the disease. The size of the opening in the vein, the depth, width and polished surface of the basin, and the temperature of the air at the time, all influence the rapidity of the coagulation of the blood, and of course, the thickness of the buffy coat.

There is a degree of conciseness and elegance in Professor Marx's style which is pleasing. The book is more like Gregory's Conspectus, in all respects, than any other work with which we are acquainted.

